# US Abortion Bans and Pregnancy-Associated Mortality

**DOI:** 10.1001/jamanetworkopen.2026.4801

**Published:** 2026-04-03

**Authors:** Hiluf Ebuy Abraha, Jeffrey Buzas, Marta Bornstein, Nansi S. Boghossian

**Affiliations:** 1 Department of Epidemiology and Biostatistics, University of South Carolina, Columbia; 2 Department of Mathematics and Statistics, University of Vermont, Burlington; 3 Department of Health Promotion, Education, and Behavior, Arnold School of Public Health, University of South Carolina, Columbia

## Abstract

**Importance:**

Few studies, mostly from the era before the *Dobbs v. Jackson Women’s Health Organization* US Supreme Court decision, have projected increases in pregnancy-related and maternal mortality in states with restrictive abortion laws. However, the association between post-*Dobbs* abortion bans and pregnancy-associated mortality remains unclear.

**Objective:**

To examine the associations of complete and 6-week abortion bans with pregnancy-associated mortality in the US.

**Design, Setting, and Participants:**

This retrospective, population-based cohort study included 14 states with abortion bans and 37 control states, including the District of Columbia, without abortion bans. US National Center for Health Statistics birth and mortality data from 2018 to 2023 were analyzed using synthetic control methods with staggered adoption, comparing ban states to their synthetic controls. Data were analyzed from July to December 2025.

**Exposure:**

State-level implementation of complete or 6-week abortion bans.

**Main Outcomes and Measures:**

The primary outcome was pregnancy-associated mortality. Secondary outcomes included pregnancy-related, maternal, and nonobstetric causes of death. Mortality ratios were examined quarterly and between preban and postban periods.

**Results:**

From 2018 to 2023, there were 22 011 131 live births and 12 993 pregnancy-associated deaths. Pregnancy-associated mortality declined by 9.8% in nonban states (54.5 [95% CI, 53.3 to 55.8] to 49.2 [95% CI, 46.5 to 52.0] deaths per 100 000 live births). In states with bans, smaller declines were observed: 2.4% in states excluding Texas (83.2 [95% CI, 80.1 to 86.3] to 81.2 [95% CI, 74.5 to 88.4] deaths per 100 000 live births) and 3.3% in Texas (54.2 [95% CI, 50.5 to 58.1] to 52.4 [95% CI, 47.4 to 57.7] deaths per 100 000 live births). Increases were largest among non-Hispanic Asian (41.0%; from 39.5 [95% CI, 27.7 to 54.7] to 55.7 [95% CI, 26.7 to 102.4] deaths per 100 000 live births) and non-Hispanic Black or African American (17.8%; from 140.2 [95% CI, 131.7 to 149.1] to 165.2 [95% CI, 144.2 to 188.3] deaths per 100 000 live births) individuals in ban states excluding Texas. Synthetic control analyses yielded an estimated difference of 5.1 (95% CI, −7.9 to 18.2) pregnancy-associated deaths per 100 000 live births, which was not statistically significant. Overall estimates for pregnancy-related mortality (−2.0; 95% CI, −11.5 to 7.5), maternal mortality (−3.0; 95% CI, −10.2 to 4.2), and nonobstetric causes (1.2; 95% CI, −7.3 to 9.7) were likewise not statistically significant. State-specific estimates were heterogeneous, but none reached statistical significance. Covariate-adjusted models yielded similar results.

**Conclusions and Relevance:**

This cohort study found that abortion bans were not associated with statistically significant overall or state-specific increases in pregnancy-associated mortality. The short postban observation window and wide CIs highlight the need for continued surveillance as more post-*Dobbs* data become available.

## Key Points

**Question:**

Are complete or 6-week abortion bans enacted after *Dobbs v. Jackson Women’s Health Organization* decision associated with changes in pregnancy-associated mortality in the US?

**Findings:**

In this cohort study including 12 993 pregnancy-associated deaths from 2018 to 2023, no significant overall increase in pregnancy-associated mortality was found in ban vs nonban states. State-specific estimates were heterogeneous, but none reached statistical significance.

**Meaning:**

This cohort study found that abortion bans were not associated with significant overall or state-specific increases in pregnancy-associated mortality in the early post-*Dobbs* period, although continued surveillance is needed, given short follow-up.

## Teaser

This cohort study examines the associations of complete and 6-week abortion bans with pregnancy-associated mortality in the US.

## Introduction

The US is facing a critical maternal health crisis, marked by approximately 669 maternal deaths—defined as deaths occurring during pregnancy or within 42 days of childbirth due to complications related to the pregnancy or its management, excluding accidents or incidental causes—each year.^1^ The US also leads other high-income countries in both infant mortality and preterm birth rates.^2,3,4^ These issues carry substantial economic and societal burdens, with the cost associated with maternal mortality estimated at $30.8 million and that of preterm birth at $13.7 billion annually.^5^ Additionally, there are persistent racial disparities in maternal health outcomes. Non-Hispanic Black or African American birthing individuals are 2 to 3 times more likely to die from pregnancy-related causes (50.3 deaths per 100 000 live births) compared with their non-Hispanic White counterparts (14.5 deaths per 100 000 live births).^1^ These figures are projected to increase with the introduction of new abortion restrictions.^6,7,8^

In June 2022, the US Supreme Court’s decision in *Dobbs v. Jackson Women’s Health Organization* overturned nearly 50 years of federal abortion protections established by *Roe v. Wade*,^9^ allowing states to impose abortion bans. As of January 2026, 13 states have implemented complete abortion bans and 28 states have imposed gestational-age restrictions, while 9 states and the District of Columbia maintain no such limitations.^10^ These bans and restrictions have sparked widespread concern about their potential impact on maternal health and racial disparities in mortality.^6,7,8,11,12^

Abortion bans can increase pregnancy-associated mortality through multiple mechanisms: by forcing the continuation of pregnancies, thereby increasing exposure to the inherent risks of pregnancy and childbirth^7,8^; by limiting the ability to terminate high-risk or nonviable pregnancies^13^; and by exacerbating existing gaps in obstetric care, especially in states with fewer maternal health resources.^14^ In addition, abortion bans may elevate pregnancy-associated mortality through nonobstetric pathways, such as worsening mental health and associated causes of death.^15^ Evidence consistently indicates that abortion is a safe and effective medical procedure^16,17^ with significantly lower risks than childbirth.^18^ However, empirical data on the association between abortion bans and pregnancy outcomes remain limited. The objective of this study was to examine the association between abortion bans and pregnancy-associated mortality in the US. We additionally assessed pregnancy-related, maternal, and nonobstetric causes of death, including state-level associations.

## Methods

This cohort study was deemed non–human participants research by the University of South Carolina institutional review board. This study followed the Strengthening the Reporting of Observational Studies in Epidemiology (STROBE) reporting guideline.

### Study Sample

This retrospective cohort study used restricted data from the National Center for Health Statistics (NCHS), including 2018 to 2023 birth and mortality records, to investigate the association between state-level post-*Dobbs* abortion restrictions and pregnancy-associated mortality. The analysis was limited to birthing individuals aged 15 to 44 years. Following NCHS guidance, we excluded individuals aged 45 years and older because the accuracy of maternal death coding declines at older ages due to increased false positives associated with the pregnancy checkbox.^19,20^

### Study Variables

The exposure was defined based on state-level abortion policy status during the study period. States were classified as having a complete abortion ban or a 6-week ban in effect, or as having no ban. For most states, exposure was considered to begin in the first quarter (Q1) of 2023 to account for the lag between policy enactment (state bans were primarily implemented immediately or within several months of the *Dobbs* decision in June 2022) and most affected pregnancies reaching delivery (approximately 5-7 months). Texas, which implemented its initial abortion ban (Senate Bill 8) in September 2021, was classified separately with the ban period beginning in Q1 2022. During the study period, exposure (ban) states included 14 states that implemented either a complete or a 6-week gestational limit (eTable 1 in Supplement 1). The control group included all remaining states and the District of Columbia, the US federal district that is not a state.

Our primary outcome was pregnancy-associated mortality, defined as the death of a woman within 365 days of the end of a pregnancy, regardless of the cause of death or the outcome of the pregnancy. It included deaths with a pregnancy checkbox indicating the decedent was pregnant at the time of death, within 42 days or up to 365 days postpartum, or deaths with an underlying cause coded using pregnancy-related *International Statistical Classification of Diseases and Related Health Problems, Tenth Revision *(*ICD-10*) codes (A34, O00-O95, O96, and O98-O99).

Within pregnancy-associated mortality, we further examined 3 categories: (1) pregnancy-related mortality, defined as deaths occurring during pregnancy or within 365 days postpartum from causes directly related to pregnancy; (2) maternal mortality, referring to deaths from pregnancy-related causes occurring during pregnancy or within 42 days postpartum (*ICD-10* codes A34, O00-O95, and O98-O99); and (3) nonobstetric causes of pregnancy-associated deaths, defined as deaths occurring during pregnancy or within 365 days postpartum from causes not directly related to pregnancy. All outcomes were expressed as mortality ratios, calculated as the number of deaths for each corresponding outcome per 100 000 live births.

Race and ethnicity were reported on US death certificates, based on information provided by a family member or another informant, or, in their absence, by funeral directors. Categories included Hispanic, non-Hispanic American Indian or Alaska Native, non-Hispanic Asian, non-Hispanic Black or African American, non-Hispanic Native Hawaiian or Other Pacific Islander, and non-Hispanic White.

### Statistical Analysis

We first compared pregnancy-associated mortality ratios per 100 000 live births before and during abortion bans by state policy group (nonban states, ban states excluding Texas, and Texas), overall and by race and ethnicity (Hispanic, non-Hispanic Asian, non-Hispanic Black or African American, and non-Hispanic White). For the overall analysis, we included all birthing individuals irrespective of race and ethnicity. For analyses stratified by race and ethnicity, we focused on birthing individuals who were Hispanic, non-Hispanic Asian, non-Hispanic Black or African American, or non-Hispanic White, as these groups had sufficient counts to produce stable descriptive mortality estimates in both the preban and postban periods. Relative changes in mortality ratios were calculated as the difference between postban and preban means divided by the preban mean. Analyses stratified by race and ethnicity were limited to descriptive comparisons; stratified modeling was not pursued because small numbers of quarterly deaths within states led to unstable state-level estimates.

We used synthetic control methods (SCM) adapted for staggered adoption to estimate policy-associated changes in mortality.^21,22^ This framework constructs a weighted combination of control units (states without bans) to approximate the counterfactual trajectory each exposed state would have followed in the absence of an abortion ban. The staggered-adoption framework was appropriate because Texas implemented its ban earlier than other states, allowing the method to account for differential timing of policy adoption.^21^

We used quarterly state-level data from 2018 to 2023 for both ban and nonban states to construct synthetic controls and estimate postban changes in mortality. The preban period was defined as 2018 to 2022 for all states except Texas, for which the preban period was 2018 to 2021. For each exposed state, we constructed a state-specific synthetic control as a weighted mean of donor states. Donor states were defined as states that had not yet implemented an abortion ban at the same point in time and were therefore eligible to contribute to the construction of the synthetic control for that exposed state. Weights were selected by the optimization algorithm to minimize differences between the exposed state and its synthetic control in preban mortality trajectories. Donor-state weight contributions used to construct the pooled synthetic counterfactual for each outcome are presented in eTable 2 in Supplement 1.

We estimated 2 SCM model specifications: an unadjusted model that balanced exclusively on preban mortality trajectories and a covariate-adjusted model that additionally balanced on 3 state-level characteristics measured during the preban period: the proportion of birthing individuals aged 25 to 34 years, the proportion of birthing individuals with a college education, and the proportion of birthing individuals identifying as non-Hispanic Black or African American. In the covariate-adjusted specification, these auxiliary variables were incorporated as additional balancing variables during construction of each state’s synthetic control. Auxiliary covariates were summarized as state-level baseline measures by calculating the mean of each covariate over the preban period for each state and were treated as time-invariant balancing variables. Model specifications were compared using pretreatment balance diagnostics, including global L2 imbalance and percentage improvement relative to uniform donor weights (eTable 3 in Supplement 1). The specification demonstrating superior pretreatment balance was selected as the primary analysis.

Both models used the full preban period to construct synthetic controls. For each exposed state, we estimated the average treatment effect on the treated (ATT) as the difference between the observed postban mortality rate and the corresponding synthetic control estimate, which represents the counterfactual mortality rate that would have occurred in the absence of an abortion ban. This estimand conditions on policy adoption and compares observed postban outcomes in exposed states with counterfactual outcomes estimated using synthetic controls. Interpretation of the ATT relies on standard SCM assumptions, including close pretreatment fit between each exposed state and its synthetic control.

State-specific ATTs were computed from the quarterly postban series, with the mean of each exposed state’s postban quarters used to produce a single state-level ATT. Overall ATTs were summarized using the multisynth average estimator,^22^ which aggregates postban effects across exposed states using a partial pooling approach.

Analyses were conducted using R software version 4.4.2 (R Project for Statistical Computing) with statistical significance set at 2-sided *P* < .05. Data were analyzed from July to December 2025.

## Results

From 2018 to 2023, there were 22 011 131 live births and 12 993 pregnancy-associated deaths (2096 Hispanic individuals [16.1%], 294 non-Hispanic American Indian or Alaska Native individuals [2.3%], 337 non-Hispanic Asian individuals [2.6%], 3653 non-Hispanic Black or African American individuals [28.1%], 214 non-Hispanic multiracial individuals [1.7%], and 6327 non-Hispanic White individuals [48.7%]). In nonban states, pregnancy-associated mortality declined by 9.8%, from 54.5 (95% CI, 53.3-55.8) to 49.2 (95% CI, 46.5-52.0) deaths per 100 000 live births between the preban period and 2023, the first full year following abortion bans. In contrast, ban states (excluding Texas) experienced only a 2.4% decline, from 83.2 (95% CI, 80.1-86.3) to 81.2 (95% CI, 74.5-88.4) deaths per 100 000 live births, while in Texas, rates fell 3.3%, from 54.2 (95% CI, 50.5-58.1) to 52.4 (95% CI, 47.4-57.7) deaths per 100 000 live births. Non-Hispanic Asian individuals in ban states (excluding Texas) experienced the largest increase (41.0%), with mortality increasing from 39.5 (95% CI, 27.7-54.7) to 55.7 (95% CI, 26.7-102.4) deaths per 100 000 live births. This was followed by non-Hispanic Black or African American individuals in ban states (excluding Texas), whose mortality increased by 17.8%, from 140.2 (95% CI, 131.7-149.1) to 165.2 (95% CI-144.2, 188.3) deaths per 100 000 live births (Table).

**Table.** Comparison of Pregnancy-Associated Mortality Ratios Before and During the Abortion Ban Period by State Group^a^

Maternal race and ethnicity by state group	Total deaths, No.	Deaths, No. (%)		Mortality ratio		
				Estimate (95% CI) per 100 000 live births		Relative change, %
		Preban period	Ban period	Preban period	Ban period	
Overall						
No bans	8421	7176 (85.2)	1245 (14.8)	54.5 (53.3 to 55.8)	49.2 (46.5 to 52.0)	–9.8
Bans (excluding Texas)^b^	3356	2815 (83.9)	541 (16.1)	83.2 (80.1 to 86.3)	81.2 (74.5 to 88.4)	–2.4
Texas	1216	1030 (84.7)	186 (15.3)	54.2 (50.5 to 58.1)	52.4 (47.4 to 57.7)	–3.3
Hispanic						
No bans	1454	1231 (84.7)	223 (15.3)	38.6 (36.5 to 40.8)	33.5 (29.3 to 38.2)	–13.2
Bans (excluding Texas)^b^	175	148 (84.6)	27 (15.4)	41.2 (34.9 to 48.4)	31.7 (20.9 to 46.1)	–23.1
Texas	467	396 (84.8)	71 (15.2)	44.1 (39.3 to 49.2)	40.3 (34.2 to 47.2)	–8.6
Non-Hispanic Asian						
No bans	252	224 (88.9)	28 (11.1)	23.9 (20.9 to 27.3)	16.0 (10.6 to 23.1)	–33.0
Bans (excluding Texas)^b^	46	36 (78.3)	10 (21.7)	39.5 (27.7 to 54.7)	55.7 (26.7 to 102.4)	41.0
Texas	39	Suppressed^c^	Suppressed^c^	26.9 (16.7 to 41.1)	Suppressed^c^	NA
Non-Hispanic Black or African American						
No bans	2153	1826 (84.8)	327 (15.2)	108.0 (103.1 to 113.1)	106.2 (95.0 to 118.4)	–1.7
Bans (excluding Texas)^b^	1236	1014 (82.1)	222 (17.9)	140.2 (131.7 to 149.1)	165.2 (144.2 to 188.3)	17.8
Texas	264	220 (83.3)	44 (16.7)	96.4 (82.9 to 111.5)	87.8 (69.9 to 108.8)	–8.9
Non-Hispanic White						
No bans	4129	3528 (85.4)	601 (14.6)	52.4 (50.7 to 54.1)	47.6 (43.8 to 51.5)	–9.0
Bans (excluding Texas)^a^	1761	1497 (84.9)	264 (15.0)	72.3 (68.7 to 76.1)	66.1 (58.3 to 74.5)	–8.6
Texas	437	377 (86.3)	60 (13.7)	59.0 (52.4 to 66.2)	60.7 (51.3 to 71.4)	2.9

Abbreviation: NA, not applicable.

^a^ Preban period was 2018 to 2022 for all states except Texas (2018 to 2021). The ban period was 2023 for all states except Texas (2022 to 2023).

^b^ Includes Alabama, Arkansas, Georgia, Idaho, Kentucky, Louisiana, Mississippi, Missouri, Oklahoma, South Dakota, Tennessee, West Virginia, and Wisconsin.

^c^ Mortality rates were suppressed when the number of deaths was 9 or fewer, per National Center for Health Statistics reporting standards.

**Figure 1.**
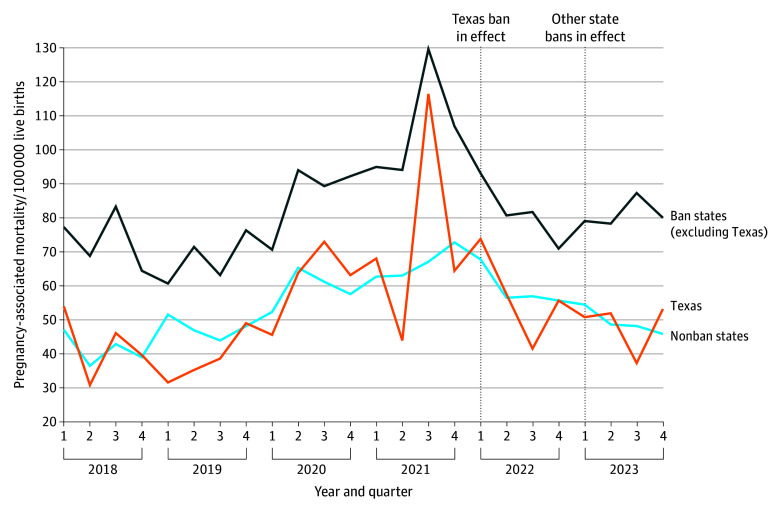
Line Graph of Descriptive Quarterly Trends in Pregnancy-Associated Mortality Quarterly pregnancy-associated mortality ratios (per 100 000 live births) from 2018 to 2023, by state policy group. These trajectories reflect unadjusted empirical trends in mortality over time.

Quarterly pregnancy-associated mortality ratios from 2018 through 2023 showed slight post-*Dobbs* divergence between nonban and ban states, with a distinct peak during the COVID-19 period (Figure 1). In the preban period, mortality ratios were consistently highest in ban states (excluding Texas). By 2023, the first full postban year, nonban states exhibited a sustained downward trajectory to fewer than 50 deaths per 100 000 live births, while rates in ban states (excluding Texas) showed a gradual upward trend. In contrast, Texas experienced irregular fluctuations after 2022.

**Figure 2.**
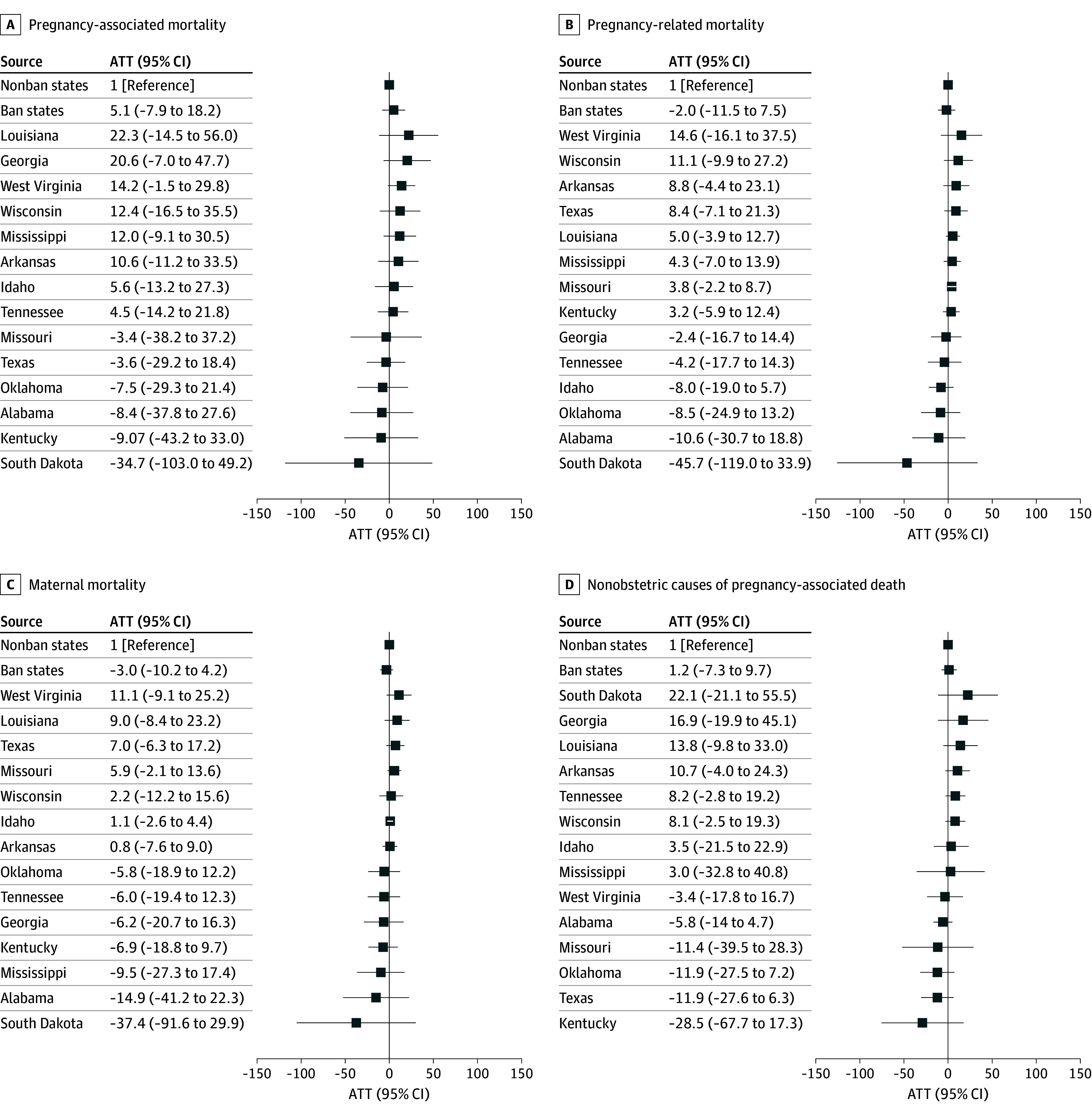
Forest Plot of Model-Based Estimates of the Association Between Abortion Bans and Mortality Outcomes From Synthetic Control Analyses Average treatment effects on the treated (ATT) estimates (per 100 000 live births) from unadjusted synthetic control models, comparing ban states with their synthetic controls in the post-*Dobbs v. Jackson Women’s Health Organization* policy period. The pre-ban period was 2018 to 2022 for all states except Texas (2018 to 2021). Ban states include Alabama, Arkansas, Georgia, Idaho, Kentucky, Louisiana, Mississippi, Missouri, Oklahoma, South Dakota, Tennessee, Texas, West Virginia, and Wisconsin. The unadjusted models consistently demonstrated closer pretreatment outcome fit than covariate-adjusted models across all outcomes, as reflected in lower global L2 values and higher percentage improvement metrics. Accordingly, the unadjusted specification was selected as the primary analysis.

Across outcomes, the unadjusted SCM demonstrated better pretreatment fit than the covariate-adjusted models, as reflected in lower global L2 imbalance and greater percentage improvement (eTable 3 in Supplement 1). Accordingly, results from the unadjusted specification are presented as the primary analysis. In this primary analysis, the estimated differences in mortality per 100 000 live births for ban states were not statistically significant, at 5.1 (95% CI, –7.9 to 18.2) pregnancy-associated deaths per 100 000 live births, –2.0 (95% CI, –11.5 to 7.5) pregnancy-related deaths per 100 000 live births, –3.0 (95% CI, –10.2 to 4.2) maternal deaths per 100 000 live births, and 1.2 (95% CI, –7.3 to 9.7) deaths of nonobstetric causes per 100 000 live births (Figure 2). State-level estimates from the SCMs varied in direction across outcomes, but none of the associations reached statistical significance (Figure 2).

In the model with auxiliary covariates, the estimated difference was 4.3 (95% CI, −13.9 to 22.6) pregnancy-associated deaths per 100 000 live births, which was not statistically significant. Estimates for overall pregnancy-related deaths, maternal deaths, and nonobstetric causes of death likewise showed no meaningful change, with ATT values of 0.6 (95% CI, –11.7 to 13.0), –2.1 (95% CI, –11.3 to 7.0), and 3.8 (95% CI, –8.0 to 15.6) deaths per 100 000 live births, respectively (eFigure in Supplement 1).

## Discussion

In this national cohort study of more than 22 million births and 12 993 pregnancy-associated deaths, pregnancy-associated mortality declined by 9.8% in states without abortion bans, compared with only a 2.4% decline in states that instituted abortion bans or limitations immediately or within several months of the *Dobbs* decision in June 2022 (ie, excluding Texas) and a 3.3% decline in Texas, which implemented its initial abortion ban in September 2021. While pregnancy-associated mortality declined overall, some groups, including non-Hispanic Asian and non-Hispanic Black or African American individuals, experienced increases in descriptive comparisons. However, in SCM analyses, abortion bans were not associated with statistically significant changes in pregnancy-associated, pregnancy-related, maternal, or nonobstetric mortality at either the overall or state-specific level. These findings should be interpreted as early and imprecise estimates, rather than definitive evidence of no effect, given the short postban observation window and wide CIs.

Pregnancy-associated mortalities peaked during the COVID-19 period, particularly in 2021. These patterns should be interpreted in the context of the pandemic, which disproportionately affected pregnant and postpartum individuals.^23^ These pandemic-related peaks likely contributed to the apparent declines in 2023 as a rebound effect rather than reflecting true improvements in health outcomes.^23,24^

Prior research projected that abortion bans would lead to higher rates of pregnancy-related and maternal mortality.^7,8^ Pre-*Dobbs* analyses similarly found that restrictive abortion policies were associated with elevated maternal mortality.^25,26,27^ These findings are consistent with global ecological evidence demonstrating that maternal mortality is lower in countries with less restrictive abortion laws.^28^ Empirical post-*Dobbs* evidence is only beginning to emerge; a 2025 study^29^ reported no significant overall difference in maternal mortality between ban and abortion-supportive states, although Texas and Louisiana exhibited higher maternal mortality ratios than the pooled mean in supportive states. The study by Nuss et al^29^ focused primarily on maternal mortality and did not examine pregnancy-associated mortality. Our analysis, which also includes nonobstetric causes of death, an increasingly prominent and potentially preventable contributor to pregnancy-associated mortality,^30,31,32,33^ adds important evidence to this emerging literature.

The absence of statistically significant policy associations in our synthetic control models is consistent with early post-*Dobbs* evidence^24^ but does not preclude the possibility that significant outcomes may emerge with longer follow-up. There are several plausible, time-dependent explanations for why national policy outcomes may not yet be detectable. First, increases in fertility following abortion restrictions, documented in ban states,^34,35^ may require additional follow-up time to translate into measurable changes in population-level mortality. Second, health system responses to bans, such as clinician relocation, clinic closures, and disruptions in referral networks,^12,14,36,37^ have already begun and are likely to continue evolving differentially across states over time, potentially amplifying or delaying impacts on obstetric care access and outcomes.

Our descriptive results stratified by race and ethnicity, including increases observed among non-Hispanic Asian and non-Hispanic Black or African American individuals in ban states (excluding Texas), highlight the importance of considering existing racial and structural inequities. Non-Hispanic Black or African American individuals already experience substantially higher pregnancy-related mortality and have long experienced a disproportionate burden of adverse maternal outcomes in the US.^1,23^ Prior to *Dobbs*, non-Hispanic Black or African American individuals also experienced higher rates of unintended pregnancy^38^ and faced greater structural barriers to obtaining abortion care,^39^ including fewer financial and logistical resources to travel out of state.^40^ These structural inequities may help contextualize the descriptive increases observed among non-Hispanic Black or African American individuals in ban states in the early post-*Dobbs* period.

This national, population-based study provides one of the first comprehensive assessments of abortion bans and pregnancy-associated mortality using linked 2018 to 2023 NCHS birth and death records. The use of synthetic controls with staggered adoption methods offers a robust approach that accounts for prepolicy differences, staggered policy adoption, and potential confounding.

### Limitations

Several important limitations warrant consideration. First, the postban observation period was short, resulting in wide CIs. Therefore, the absence of statistically significant associations should be interpreted cautiously as early, imprecise estimates rather than evidence of no change. Second, state-level abortion policy classification may not fully capture individual-level exposure. Following *Dobbs*, overall abortion provision increased nationally, driven by expansions in abortion-supportive states and substantial increases in states adjacent to ban states.^41,42^ As a result, exposure to abortion bans is likely heterogeneous across individuals residing in ban states, which may attenuate early, population-level estimates of policy outcomes and further obscure subgroup-specific risks. Third, there may be potential misclassification due to missing data on gestational age and postpartum timing at death, although such errors likely occurred in both directions, minimizing bias. Fourth, misclassification from the pregnancy checkbox also remains a known issue, particularly for older decedents and postpartum deaths,^20^ which may inflate or underestimate true mortality. Although restricting analyses to birthing individuals aged 15 to 44 years mitigated some false positives, residual misclassification is possible. Furthermore, NCHS suppresses death counts of 9 or fewer for confidentiality, which prevented us from reporting mortality ratios for some racial and ethnic groups with very small numbers of deaths.

## Conclusions

This cohort study found that abortion bans were not associated with statistically significant overall or state-specific increases in pregnancy-associated, pregnancy-related, maternal, or nonobstetric mortality. The wide CIs and short postban observation period underscore the importance of continued surveillance. As additional data from the NCHS become available, future analyses with longer follow-up periods will be critical to assess whether effects emerge over time.

## Notes

Supplement 1. **eTable 1.** State abortion restrictions and ban exposure start quarter **eTable 2.** Weights for each state that contributed to the synthetic control of each outcome **eTable 3.** Goodness-of-fit diagnostics for synthetic control models **eFigure.** Model-based estimates of the association between abortion bans and mortality outcomes from covariate-adjusted synthetic control analyses

Supplement 2.
Data Sharing Statement
