# Supplementary material for: US Abortion Bans and Pregnancy-Associated Mortality
Source: JAMA Netw Open. 2026 Apr 3;9(4):e264801. doi: 10.1001/jamanetworkopen.2026.4801 (PMC13049493; doi:10.1001/jamanetworkopen.2026.4801)
Supplement: Supplement 1. — eTable 1. State abortion restrictions and ban exposure start quarter eTable 2. Weights for each state that contributed to the synthetic control of each outcome eTable 3. Goodness-of-fit diagnostics for synthetic control models eFigure. Model-based estimates of the association between abortion bans and mortality outcomes from covariate-adjusted synthetic control analyses [file jamanetwopen-e264801-s001.pdf]

Supplemental Online Content

Abraha HE, Buzas J, Bornstein M, Boghossian NS. US abortion bans and pregnancy-associated mortality. *JAMA Netw Open*. 2026;9(4):e264801. doi:10.1001/jamanetworkopen.2026.4801

eTable 1. State abortion restrictions and ban exposure start quarter

eTable 2. Weights for each state that contributed to the synthetic control of each outcome

eTable 3. Goodness-of-fit diagnostics for synthetic control models

eFigure. Model-based estimates of the association between abortion bans and mortality outcomes from covariate-adjusted synthetic control analyses

This supplemental material has been provided by the authors to give readers additional information about their work.

© 2026 Abraha HE et al. *JAMA Network Open*.

eTable1. State Abortion Restrictions and Ban Exposure Start Quarter

State	Policy summary*	Implementation date	Ban exposure start quarter
Alabama	Prohibits abortion at any gestation; exception if deemed medically necessary to preserve the pregnant person's life	6/24/22	Q1 2023
Arkansas	Prohibits abortion at any gestation; exception to save the pregnant person's life	6/24/22	Q1 2023
Georgia	Bans abortion after 6 weeks' gestation; exceptions for threats to life or health, lethal fetal anomalies, or pregnancies from rape/incest (up to 20 weeks)	11/1/22	Q1 2023
Idaho	Prohibits abortion at any gestation; exceptions to save the pregnant person's life or for rape/incest reported in the first trimester	8/25/22	Q1 2023
Kentucky	Prohibits abortion at any gestation; exceptions for life-threatening conditions or preventing major organ impairment	6/24/22	Q1 2023
Louisiana	Prohibits abortion at any gestation; exceptions for life-threatening conditions, severe permanent injury, or lethal fetal anomaly	6/24/22	Q1 2023
Mississippi	Prohibits abortion at any gestation; exceptions to save the pregnant person's life or for rape reported to law enforcement	6/27/22	Q1 2023
Missouri	Prohibits abortion at any gestation; exception for medical emergencies	6/24/22	Q1 2023
Oklahoma	Initially bans abortion after 6 weeks (May 2022); expanded to total ban by June 2022; exception to save the pregnant person's life	05/03/22	Q1 2023
South Dakota	Prohibits abortion at any gestation; exception to save the pregnant person's life	06/24/22	Q1 2023
Tennessee	Prohibits abortion at any gestation; limited medical emergency exceptions	8/25/22	Q1 2023
Texas	6-week limit enacted September 2021; total ban August 2022; exceptions for life or serious health threats	09/01/21	Q1 2022
West Virginia	Prohibits abortion at any gestation; exceptions for nonviable fetus, ectopic pregnancy, medical emergency, or certain rape/incest cases (up to 14 weeks)	08/13/22	Q1 2023
Wisconsin	Nearly all abortions ceased following the June 2022 Dobbs decision due to interpretation of an 1849 law; the legal status was clarified in July 2023 (20-week limit)	06/24/22	Q1 2023

* Policy summary was based on the following sources: Center for reproductive rights, https://reproductiverights.org/maps/abortion-laws-by-state/ (Accessed May 29, 2025) and Guttmacher Institute, https://www.guttmacher.org/state-policy/explore/state-policies-abortion-bans (Accessed April 05, 2025).

eTable 2. Weights for each state that contributed to the synthetic control of each outcome.

© 2026 Abraha HE et al. *JAMA Network Open*.

Donor state	Weights			
	**Pregnancy-associated mortality**	**Pregnancy-related mortality**	**Maternal Mortality**	**Non-obstetric related mortality**
Florida	0.3260	0.5550	0.4680	-
Iowa	0.1220	0.0270	0.0847	0.0168
Massachusetts	0.0792	-	-	-
South Carolina	0.0760	0.0674	-	-
Virginia	0.0606	-	-	-
Colorado	0.0519	0.0218	0.0196	0.0523
New Mexico	0.0506	0.0571	0.0355	0.0325
Alaska	0.0454	0.0152	-	0.0273
Hawaii	0.0415	0.0266	0.0204	0.0474
Minnesota	0.0330	-	-	0.0290
Nevada	0.0328	0.0226	0.0314	-
North Carolina	0.0239	0.0389	0.0387	0.0420
Vermont	0.0195	-	-	0.0504
Montana	0.0179	-	-	-
Rhode Island	-	0.0357	0.0663	-
Wyoming	-	-	0.0160	-
North Dakota	-	-	-	-
District of Columbia	-	-	-	-
Maine	-	0.0222	0.0116	0.0109
New York	-	0.0278	-	-
Oregon	-	0.0133	-	0.0412
California	-	-	0.0138	-
Illinois	-	0.0384	-	-
Arizona	-	-	-	0.0166
Delaware	-	-	0.0144	-
Nebraska	-	0.0191	-	0.0545
New Hampshire	-	-	0.0835	-
Kansas	-	-	-	0.0121
Washington	-	-	0.0732	0.0470
Michigan	-	-	-	0.0126
Maryland	-	-	-	0.2240
Ohio	-	-	-	0.1250
Utah	-	-	-	0.0717
New Jersey	-	-	-	-
Connecticut	-	-	-	0.0395
Pennsylvania	-	-	-	-
Indiana	-	-	-	-

Donor weights represent each control state's contribution to the pooled synthetic counterfactual used to estimate the average treatment effect across the 14 ban states.

© 2026 Abraha HE et al. *JAMA Network Open*.

eTable 3. Goodness-of-fit diagnostics for synthetic control models

Outcomes	Model	Global L2	Scaled Global L2	Percent improvement (Global)	Individual L2	Scaled individual L2	Percent improvement (Individual)
Pregnancy-associated mortality	Crude	4.9	0.191	80.9	24.2	0.557	44.3
	Adjusted	8.6	0.336	66.4	29.8	0.686	31.4
Pregnancy-related mortality	Crude	4.8	0.264	73.6	18.2	0.617	38.3
	Adjusted	7.1	0.389	61.1	21.7	0.734	26.6
Maternal mortality	Crude	4.5	0.346	65.4	15.4	0.635	36.5
	Adjusted	6.9	0.527	47.3	19.2	0.792	20.8
Non-obstetric related mortality	Crude	2.0	0.178	82.2	15.8	0.624	37.6
	Adjusted	6.3	0.550	45.0	20.0	0.792	20.8

Synthetic Control Models

Data structure and preparation

Synthetic control analyses were conducted using a quarterly panel of 51 units (50 states and the District of Columbia) observed from 2018 through 2023, yielding 1224 state-quarter observations. For each outcome – pregnancy-associated mortality, pregnancy-related mortality, maternal mortality, and non-obstetric causes of pregnancy-associated death – we calculated quarterly mortality ratios (per 100 000 live births). All synthetic control models were estimated using a fixed random seed (set.seed = 123456) to ensure reproducibility.

Pooling specification

For each treated state, a synthetic control was constructed to match its pre-treatment outcome trajectory using a weighted combination of donor states that had not yet implemented abortion bans. State-specific treatment effects were calculated by comparing observed post-ban outcomes with the corresponding synthetic control for each treated state and averaging differences across post-ban quarters.

In contrast, the overall treatment effect was summarized using the multisynth "Average" estimator with the package's default pooling parameter, which partially pools information across treated states to stabilize the aggregate estimate while allowing for heterogeneous state-level effects

Assumptions, model specifications, and diagnostics

Interpretation of estimated effects using synthetic control methods relies on the assumption that each treated state's synthetic control provides a valid approximation of its counterfactual outcome trajectory in the absence of the policy, conditional on close pre-treatment fit.

We assessed pre-treatment balance and model fit using diagnostics implemented in the *augsynth* package for augmented synthetic control models. Crude models correspond to synthetic controls constructed using lagged outcomes only, whereas adjusted models additionally incorporated auxiliary covariates as balancing variables. Auxiliary covariates were summarized as state-level baseline measures by averaging each covariate over the pre-ban period for each state and treated as time-invariant balancing variables in the synthetic control models. These diagnostics quantify how closely each synthetic control reproduces the exposed state's pre-treatment outcomes and

© 2026 Abraha HE et al. *JAMA Network Open*.

how much optimized SCM weights improve balance relative to uniform donor weighting. All diagnostics were calculated over the pre-treatment period (2018–2022 for all states, except Texas where the pre-period was 2018–2021).

Specifically, global L2 imbalance is the Euclidean (L2) distance between the exposed state's and synthetic control's pre-treatment outcome series; scaled global L2 normalizes this value by the magnitude of the exposed state's pre-treatment outcomes; and percent improvement from uniform global weights reports the reduction in global L2 achieved by optimized weights relative to uniform weights. At the predictor level, individual L2 imbalance and scaled individual L2 assess balance on the predictors used in the optimization – lagged outcomes in the crude model and lagged outcomes plus auxiliary covariates in the adjusted model. Percent improvement from uniform individual weights summarizes the relative improvement in predictor-level balance.

Interpretation of diagnostics

Across outcomes, crude models consistently demonstrated closer pre-treatment outcome fit than covariate-adjusted models. Non-obstetric mortality exhibited the best global fit (Global L2 = 2.0; 82.2% improvement), followed by pregnancy-associated mortality (Global L2 = 4.9; 80.9% improvement) and pregnancy-related mortality (Global L2 = 4.8; 73.6% improvement). Maternal mortality showed comparatively lower improvement (Global L2 = 4.5; 65.4% improvement), reflecting more modest gains from optimized weighting.

© 2026 Abraha HE et al. *JAMA Network Open*.

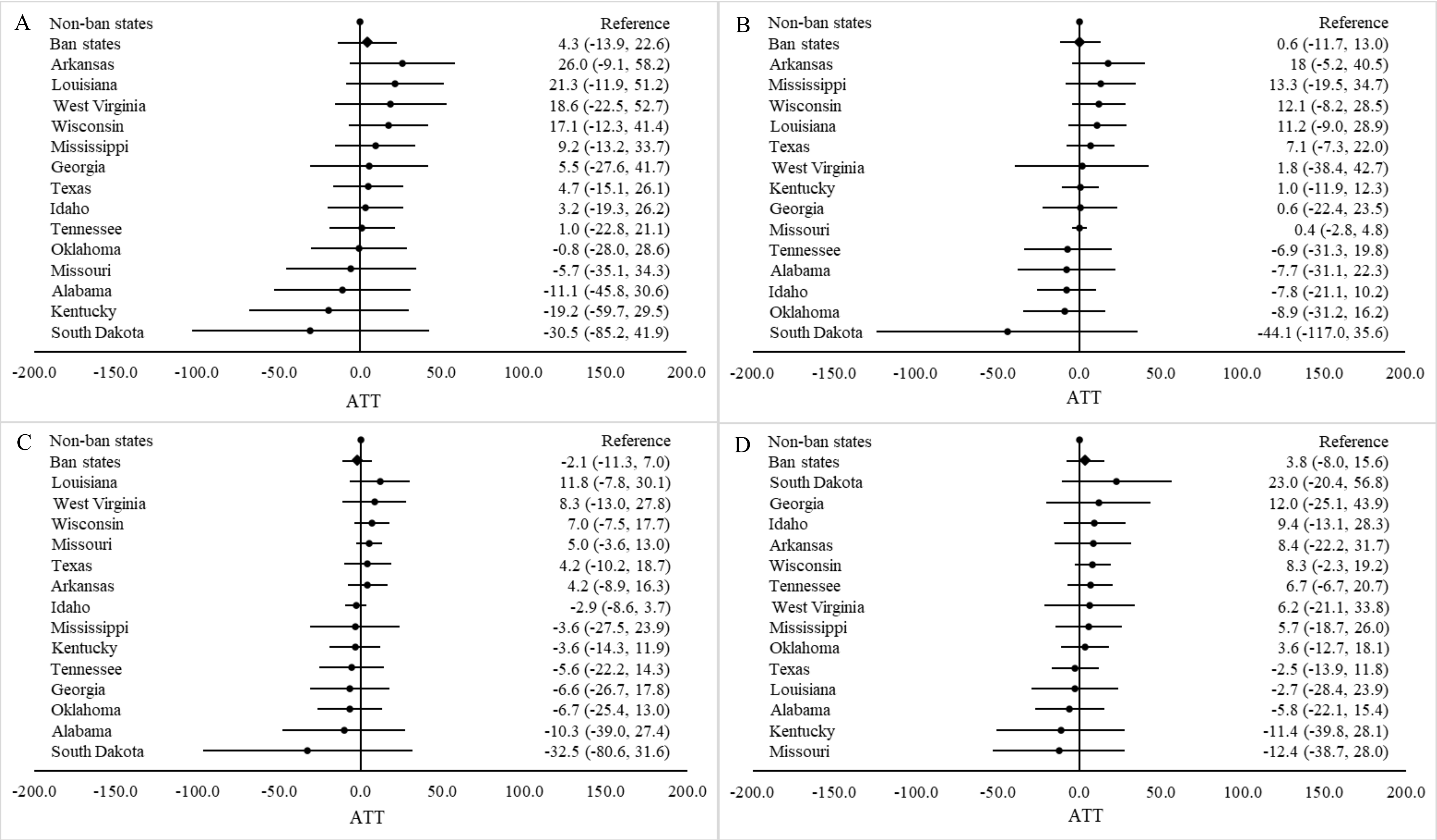

eFigure. Model-based estimates of the association between abortion bans and mortality outcomes from covariate-adjusted synthetic control analyses.

© 2026 Abraha HE et al. *JAMA Network Open*.

Average treatment effects on the treated (ATT) estimates (per 100 000 live births) from covariate-adjusted synthetic control models, comparing ban states with their synthetic controls in the post-Dobbs policy period for (A) pregnancy-associated mortality, (B) pregnancy-related mortality, (C) maternal mortality, and (D) non-obstetric causes of pregnancy-associated death.
Covariates included the proportion of birthing individuals aged 25-34 years, the proportion with a college education, and the proportion who are non-Hispanic Black. The pre-ban period was 2018–2022 for all states except Texas (2018–2021). Ban states include Alabama, Arkansas, Georgia, Idaho, Kentucky, Louisiana, Mississippi, Missouri, Oklahoma, South Dakota, Tennessee, Texas, West Virginia, and Wisconsin.

© 2026 Abraha HE et al. *JAMA Network Open*.
