# Supplementary material for: US Abortion Bans and Pregnancy-Associated Mortality
Source: JAMA Netw Open. 2026 Apr 3;9(4):e264801. doi: 10.1001/jamanetworkopen.2026.4801 (PMC13049493; doi:10.1001/jamanetworkopen.2026.4801)
Supplement: Supplement 2. — Data Sharing Statement [file jamanetwopen-e264801-s002.pdf]

Data Sharing Statement

Abraha. US Abortion Bans and Pregnancy-Associated Mortality. *JAMA Netw Open*. Published April 03, 2026. doi:10.1001/jamanetworkopen.2026.4801

Data
Data available: No

Additional Information
Explanation for why data not available: The data can be obtained via a DUA from NCHS.
